# Fostering efficacy of anti-PD-1-treatment: Nivolumab plus radiotherapy in advanced non-small cell lung cancer - study protocol of the FORCE trial

**DOI:** 10.1186/s12885-019-6205-0

**Published:** 2019-11-08

**Authors:** Farastuk Bozorgmehr, Adriane Hommertgen, Johannes Krisam, Felix Lasitschka, Jonas Kuon, Martin Maenz, Peter E. Huber, Laila König, Meinhard Kieser, Juergen Debus, Michael Thomas, Stefan Rieken

**Affiliations:** 1 Department of Thoracic Oncology, Thoraxklinik at University Hospital of Heidelberg, Röntgenstraße 1, 69126 Heidelberg, Germany; 2 Translational Lung Research Center Heidelberg TLRCH, Member of the German Center for Lung Research DZL, Im Neuenheimer Feld 156, 69120 Heidelberg, Germany; 3 Department of Radiation Oncology, University Hospital of Heidelberg, Im Neuenheimer Feld 400, 69120 Heidelberg, Germany; 4 German Cancer Research Center, Abteilung für Molekulare Radioonkologie, Im Neuenheimer Feld 280, 69120 Heidelberg, Germany; 5 Heidelberg Institute of Radiation Oncology HIRO, Im Neuenheimer Feld 280, 69120 Heidelberg, Germany; 6 Institute of Medical Biometry and Informatics, University Hospital of Heidelberg, Im Neuenheimer Feld 130.3, 69120 Heidelberg, Germany; 7 University Hospital of Heidelberg, Institute of Pathology, Im Neuenheimer Feld 430, 69120 Heidelberg, Germany; 8 AIO-Studien gGmbH, Berlin, Germany

**Keywords:** Non-small cell lung cancer, Immunotherapy, Radioimmunotherapy, Abscopal effect, PD-1, Nivolumab, Palliative radiotherapy

## Abstract

**Background:**

Hypofractionated palliative radiotherapy for metastatic lung cancer patients is frequently used in order to ease pain, to increase bone stability, to treat local mass effects, or to prolong progression-free survival at critical sites. Recently introduced, immunotherapy for patients with non-squamous non-small cell lung carcinoma (NSCLC) has significantly improved outcome in this cohort. Preclinical and early clinical data suggest that the combination of photon radiation with programmed death-1 (PD-1) targeting immunotherapies may promote a strong and durable immune response against tumor manifestations both within and beyond radiation targets.

**Methods/design:**

In the present prospective, two-group, non-randomized, open-label phase II trial, 130 patients with stage IV non-squamous NSCLC in 2nd-line or 3rd-line treatment will be included. 65 patients with a clinical indication for palliative radiotherapy to non-cerebral/non-pulmonary metastatic sites will receive 240 mg nivolumab followed by palliative radiotherapy with 5 × 4 Gray (Gy) = 20 Gy photon radiation, which will be initiated within 72 h after first nivolumab administration (Group A). 65 patients without an indication for radiotherapy will only receive nivolumab (Group B). Nivolumab will be further administered every two weeks in both groups and will be continued until progression and loss of clinical benefit or until occurrence of limiting toxicities.

The primary endpoint will be the objective response rate (ORR) according to response evaluation criteria in solid tumors (RECIST) 1.1. Secondary endpoints will be progression-free survival (PFS) according to RECIST 1.1, overall survival, descriptive subgroup analyses according to PD-L1 expression, toxicity and quality of life. Since response patterns following immunotherapies differ from those after conventional cytostatic agents, both objective response rate and progression-free survival will additionally be assessed according to immune-related RECIST (irRECIST) criteria.

**Discussion:**

The FORCE study will prospectively investigate response rates, progression-free and overall survival (OS), and toxicity of nivolumab with and without hypofractionated palliative radiotherapy in a group of 130 patients with metastatic non-small cell lung cancer (non-squamous histology) in 2nd-line or 3rd-line treatment. This trial will contribute prospective data to the repeatedly published observation that the combination of hypofractionated photon radiotherapy and medical immunotherapy is not only safe but will also promote antitumoral immune responses.

**Trial registration:**

Clinicaltrials.gov identifier: NCT03044626 (Date of initial registration: 05 January 2017).

Eudra-CT Number: 2015–005741-31 (Date of initial registration: 18 December 2015).

## Background

Despite continuously evolving treatment innovations, NSCLC remains to be one of the most lethal cancer diagnoses. In metastatic patients, radiotherapy is frequently administered for several reasons, for instance to ease tumor pain, to increase bone stability or to mitigate localized disease symptoms and conditions from mass effects to tumor infiltrations such as bleeding, ulceration or organ compressions [1]. Recently, immunotherapies have been introduced as new treatment modalities aiming for the disinhibition of the natural antitumoral immune response. Significant benefits translating into tremendously improved progression-free survival and overall survival rates have been described for patients with stage IV renal cell carcinoma and melanoma and lately also for patients with squamous or non-squamous NSCLC [2–5]. Among the many potential molecular structures that may be targeted pharmacologically, treatments directed against the PD-1/PD-L1 immune checkpoint have improved survival at the cost of only modest toxicity for NSCLC patients in both 1st- and 2nd- line treatment situations. However, response rates range around only 20% in previously treated patients, and also frontline administration of PD-1 inhibitors results in no tumor response in approximately half of the treated patients [4, 6, 7]. In order to identify patients more likely to respond to PD-1 blockade the expression of PD-L1 on tumor cells has been introduced as a biomarker. The utility of PD-L1 as a predictive biomarker, however, is still under debate, and alternatives such as tumor mutation burden (TMB) are now taken into account [7–9].

Radiotherapy has been the predominant local treatment for tumor metastases for more than five decades and occasionally an interplay between photon radiation and tumor-directed immune responses has been described [10–13]. Specifically, photon radiation to one metastatic site has been observed to elicit a response to non-irradiated tumor sites – commonly referred to as the abscopal effect, which was first described in 1953 [14]. Radiation is known to induce immunogenic cell death, which is a unique expression pattern of cell damage-derived proteins from both tumor and stromal cells that may activate the immune system and promote the recognition of tumor-associated/−specific proteins elsewhere in the body [10, 15, 16].

However, when radiation is applied as a sole treatment modality, this phenomenon is soon suppressed by regulatory signalling pathways that inhibit auto−/ tumor-immune responses within and outside the tumor microenvironment [13, 17, 18]. Thus, the clinical observation of any abscopal effect with radiation alone has always been a rare finding. With the advent of agents that target PD-1/PD-L1 and therefore disinhibit tumor-directed immune responses, the potential of inducing an abscopal effect through combined radio-immunotherapies has gained renewed attention. Interestingly, a secondary analysis of a clinical landmark trial has identified 98 patients, who had received photon radiotherapy prior to immunotherapy [19]. These patients showed significantly improved PFS and OS – irrespective of the expression of PD-L1. This finding has – once again – nourished the hope that the combination of pharmacological disinhibition of the immune system through medical immunotherapies and tumor-antigen-exposing photon radiation may be a beneficial combination. However, to date results from prospective clinical trials investigating this hypothesis in lung cancer patients are not available.

## Methods/design

### Study design

**Fig. 1:**
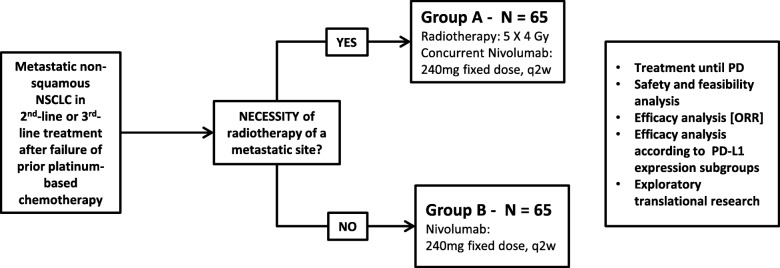
FORCE patient allocation and efficacy analysis strategy

This is an interventional two-group, non-randomized, open-label phase II trial (Fig. 1) [20].

Patients with necessity of radiotherapy of a metastatic site (e.g. bone) will be assigned to study group A. Patients without the necessity of radiotherapy will be assigned to study group B.

### Study setting

The FORCE trial is a multicenter trial recruiting patients from 16 sites across Germany. A full list of sites can be obtained at clinicaltrials.gov (NCT03044626). Recruitment started in March 2017 (First Patient In) and will be completed in December 2019. As of August 2019, 104 patients have been screened, of whom 94 have been included in the trial. Overall, data from 100 participants (50 patients per treatment arm) are expected to be available for statistical analysis.

### Study objectives

#### Primary objective

The primary objective is to investigate clinical efficacy of a nivolumab-radiotherapy combination treatment.

#### Secondary objectives

Secondary objectives are to collect information on feasibility, safety and tolerability of nivolumab by measurement of incidence and severity of adverse events (AEs) and specific laboratory abnormalities in all treated subjects by treatment strata and groups. Additionally, further efficacy data will be collected in patients without necessity of radiotherapy as well as information on individual, patient reported and investigator-assessed quality of life.

#### Exploratory objectives

Exploratory objectives aim to investigate potential predictors of response to nivolumab in conjunction with radiotherapy. To this end, tissue collection and blood sampling will be performed before and whilst course of disease/treatment. Exploratory analysis on blood samples and tissue will be performed to search for markers of immune response under radio-immunotherapy.

The following exploratory objectives will be investigated:

- Biomarker assessment of tumor tissue by immunohistochemistry (IHC) beyond PD-L1
- Phenotypical analysis of lymphocytes
- Functional analysis of T-cells
- Analysis of T-cell receptor specificities
- Soluble pro- and anti-inflammatory markers

Analysis of biomarker data will include correlation with clinical phenotype and tumor PD-L1 expression.

Furthermore, to address the role of radiotherapy in the context of immune modulation, several aspects of radiation planning and treatment will be explored. This includes both the location and composition of radiation targets and the anatomical profile of abscopally responding lesions. Therefore, treatment-related aspects characterizing the irradiated targets and abscopally responding target lesions will be documented by the treating radiation oncologist and radiologist. Documentation of these aspects will help to substantiate the phenomenon of radiation-induced abscopal effects and to improve the option of radiation triggered systemic response through identification and possible prediction of both eligible targets for irradiation and probable lesion of abscopal response.

### Characteristics of participants

One hundred thirty patients with metastatic non-small cell lung cancer (non-squamous histology) in 2nd-line and 3rd-line treatment will be included. Patients who might be eligible for this clinical trial will be approached and asked to participate as they come into the clinic.

Key inclusion criteria contain age ≥ 18 years, non-squamous NSCLC with available (recent or archival) paraffin-embedded tissue blocks for PD-L1-expression evaluation, failure after platinum-based 1st-line or 2nd-line treatment, and adequate clinical performance (ECOG 0–1). Patients in group A must present with a clinical indication for palliative radiotherapy to non-cerebral/non-pulmonary metastatic sites with the additional presence of at least one non-irradiated measurable site of disease. Patients in group B must not present with any indication for radiotherapy. Key exclusion criteria contain ongoing systemic steroid treatment (> 10 mg/day of prednisone equivalents), prior immunotherapy, an active or recent history of a known or suspected autoimmune disease, or any medical conditions conflicting with the study interventions. Patients with brain metastases requiring local or corticosteroidal treatment cannot be included. For a full list of the inclusion an exclusion criteria see Table 1.

**Table 1:** Complete list of inclusion and exclusion criteria

Inclusion criteria	
• Written informed consent and any locally-required authorization (EU Data Privacy Directive in the EU) obtained from the subject prior to performing any protocol-related procedures, including screening evaluations. • Subject is willing and able to coxmply with the protocol for the duration of the study including undergoing treatment and scheduled visits and examinations including follow up. • Age ≥ 18 years at time of study entry. • ECOG performance status 0–1. • Patients with measurable disease (at least one uni-dimensionally measurable target lesion by CT-scan or MRI) according to RECIST 1.1 are eligible. For patients in group A, non-measurable and measurable lesions may be chosen for irradiation. However, in order to allow for evaluation of abscopal effects, patients in group A must have at least one measurable lesion beside the lesion planned to be irradiated. Lesions planned to be irradiated may not be defined as a measurable target lesion. Radiographic tumor assessment must be performed within 28 days before initiation of study treatment. • Target Lesions may be located in a previously irradiated field if there is documented (radiographic) disease progression in that site. • Patients with metastatic non-squamous NSCLC in 2nd-line and 3rd-line treatment and a) no necessity of radiotherapy or b) the necessity of radiotherapy of a metastatic bone lesion or soft tissue lesion. • Patients with intrathoracic metastases or intrathoracic progressive disease will be included if radiotherapy of the lung parenchyma is NOT required. • Subjects with symptomatic brain metastases are eligible if metastases have been treated and treatment has been completed at least 12 weeks before inclusion in this study for group B and 2 weeks for group A. Moreover, there must be no MRI evidence of progression within 28 days prior to the first dose of nivolumab administration. There must also be no requirement for immunosuppressive doses of systemic corticosteroids (> 10 mg/day prednisone equivalents) for at least 2 weeks prior to study drug administration. Patients with stable/asymptomatic brain metastases that do not require local therapy with irradiation (whole brain irradiation or stereotactic brain irradiation) can be included. In ambiguous cases, consultation with the LKP or his/her delegate is advised. • An FFPE tumor tissue block (archival or recent) or a minimum of 15 unstained slides of tumor sample must be available for biomarker (PD-L1) evaluation. • Prior therapies and surgeries are allowed if completed 2 weeks for minor surgery (group A and B) or 12 weeks for any previous radiotherapy for group B, respectively prior to start of treatment and patient recovered from toxic effects. For group A, any prior radiotherapy not involving the lungs must be completed 2 weeks prior to start of treatment. A prior radiotherapy involving the lungs must be completed 12 weeks prior to start of treatment. • Subjects must have recovered from the effects of major surgery or significant traumatic injury at least 14 days before the first dose of study treatment. • Adequate blood count, liver-enzymes, and renal function (obtained no later than 14 days prior to start of treatment): • Women of childbearing potential (WOCBP) must use appropriate method(s) of contraception and must have a negative serum pregnancy test within 24 h prior to the start of nivolumab. • Men who are sexually active with WOCBP must use any contraceptive method with a failure rate of less than 1% per year.	
Exclusion criteria	
• Previous malignancy (other than NSCLC), which either progresses or requires active treatment. • Subjects with previous malignancies (except non-melanoma skin cancers, and the following in situ cancers: bladder, gastric, colon, cervical/dysplasia, endometrial, melanoma, or breast) are excluded unless a complete remission was achieved at least 2 years prior to study entry AND no additional therapy is required or anticipated to be required during the study period. • Brain metastases mandating active treatment in terms of irradiation (whole brain irradiation or stereotactic brain irradiation). • Known activating EGFR mutation or a known ALK translocation. • Prior therapy with anti-tumor vaccines or other immuno-stimulatory antitumor agents. • Patients with interstitial lung disease. • Any previous treatment with an anti-PD-1, anti-PD-L1, anti-PD-L2, anti-CTLA-4 antibody, or any other antibody or drug specifically targeting T cell co-stimulation or immune checkpoint pathways. • All toxicities attributed to prior anti-cancer therapy other than alopecia and fatigue must have resolved to grade 1 (CTCAE version 4) or baseline before administration of study drug. • Patients should be excluded if they have an active, known or suspected autoimmune disease. Subjects are permitted to enroll if they have vitiligo, type I diabetes mellitus, residual hypothyroidism due to autoimmune condition only requiring hormone replacement, psoriasis not requiring systemic treatment, or conditions not expected to recur in the absence of an external trigger. • Patients should be excluded if they have a condition requiring systemic treatment with either corticosteroids (> 10 mg daily prednisone equivalents) or other immunosuppressive medications within 14 days of study drug administration. • Patients should be excluded if they are positively tested for hepatitis B virus surface antigen (HBV sAg) or hepatitis C virus ribonucleic acid (HCV antibody) indicating acute or chronic infection. • Patients should be excluded if they have known history of testing positive for human immunodeficiency virus (HIV) or known acquired immunodeficiency syndrome (AIDS). • History of severe hypersensitivity reactions to other monoclonal antibodies or any excipient. • Female subjects who are pregnant, breast-feeding or male or female patients of reproductive potential, who are not employing an effective method of birth control (failure rate of less than 1% per year) • Receipt of the last dose of anti-cancer therapy (chemotherapy, immunotherapy, endocrine therapy, targeted therapy, biologic therapy, tumor embolization, monoclonal antibodies, other investigational agent) ≤ 14 days prior to the first dose of study treatment • Any other serious or uncontrolled medical disorder, active infection, physical examining, laboratory finding, altered mental status, or psychiatric condition that, in the opinion of the investigator, would limit a subject’s ability to comply with the study requirements, substantially increase risk to the subject, or impact the interpretability of study results • History of solid organ or tissue transplantation including allogenic hematopoietic stem cell transplantation • Previous enrollment in the present study • Involvement in the planning and/or conduct of the study (applies to both BMS staff and/or staff of sponsor and study site) • Patient, who might be dependent on the sponsor, site or the investigator • Patient, who has been incarcerated or involuntarily institutionalized by court order or by the authorities § 40 Abs. 1 S. 3 Nr. 4 AMG	

### Study procedures

This study will enroll patients with metastatic non-squamous NSCLC suitable for 2nd-line and 3rd-line treatment. Study subject may have a necessity for radiotherapy of a metastatic site (study group A) or not (group B).

This is an open-label, two-group study. During screening and after written informed consent, patients will be stratified to either the radiotherapy/nivolumab treatment (group A) or nivolumab only treatment (group B), depending on necessity of radiation of a metastatic site.

An overview of all study procedures is presented in Table 2. For each patient enrolled, an electronic case report form (eCRF) must be completed by the principal investigator or authorized delegate from the study staff. This also applies to records for those patients who fail to complete the study. If a patient withdraws from the study, the reason must be noted in the eCRF. Subjects who are permanently discontinued from the study medication will be followed for safety unless consent is withdrawn or the subject is lost to follow-up or enrolled in another clinical study. All subjects will be followed for survival. Subjects who decline to return to the site for evaluations will be offered follow-up by phone every 3 months as an alternative.

**Table 2:** Schedule of assessments

Procedure/Point in Time		Screening	Treatment				Follow-up	
		Inclusion	Cycle 1		Every further cycle (q2w)	End of treatment	Safety follow-up	Survival follow-up
			Day 1	Day 8				
Informed consent, eligibility criteria, demographics, medical and disease history		x						
FFPE tumor tissue (PD-L1)		x						
Vital signs, physical examination, ECOG		x	x	x	x	x	x	
Hematology, serum chemistry, liver function tests		x	x	x	x	x	x	
HBV, HCV		x						
Thyroid function test		x	x		x ^a^	x	x	
Pregnancy test		x			x ^a^			
AEs/SAEs		x	continuously				x	
Concomitant medication		x	continuously				x	x
QoL		x	x		x ^b^	x	x	x
Biomarker sample		x		x	x	x		
Radiographic tumor assessments		x			x ^a^		x	x
Survival information, subsequent therapies								x
Group A	Nivolumab		x		x			
	Radiotherapy		x	(x)				
Group B	Nivolumab		x		x			

^a^: every 3rd cycle (every 6 weeks)

^b^: every other cycle (every 4 weeks)

Data management and data quality assurance are conducted following the Standard Operational Procedures of the Institut für Klinische Forschung (IKF) (Frankfurt, Germany).

Treatment emergent adverse events (AEs) according to common terminology criteria for adverse events (CTCAE) version 4.03 will be recorded in the eCRF using a recognized medical term or diagnosis that accurately reflects the event. Adverse events will be assessed by the investigator for severity, relationship to the investigational product, possible etiologies, and whether the event meets criteria of a serious adverse event (SAE) and therefore requires immediate notification to the CRO. AEs and SAEs will be recorded during the entire study duration, including the regular 30 day safety follow-up period after the end-of-treatment (EOT) visit. Subsequently, subjects will be followed for ongoing study treatment-related adverse events until resolved, return to baseline or deemed irreversible, until lost to follow-up, or withdrawal of study consent. Furthermore, only new and ongoing SAEs deemed related to study treatment will be collected and recorded for an additional 70 days. The investigator is responsible for ensuring that all adverse events observed by the investigator or reported by patient are properly captured in the patients’ medical records. During the course of the study all AEs and SAEs should be proactively followed up for each subject. Every effort should be made to obtain a resolution for all events, even if the events continue after discontinuation/study completion.

#### Immunotherapy

Nivolumab will be given every two weeks at a dose of 240 mg to be administered as a 60 min IV infusion. Treatment regimen depends on study group: in study group A, nivolumab will be given on day 1 of the first cycle and continued to be given every two weeks. The first fraction of radiotherapy has to be delivered within 72 h after cycle 1 day 1.

In study group B, nivolumab will be given on day 1 of the first cycle and continued to be given every two weeks.

In both groups, nivolumab treatment will be continued until progression or until limiting toxicities occur.

Nivolumab administration will be delayed in case of any AE, laboratory abnormality or intercurrent illness which, in the judgment of the investigator, warrants delaying the dose of study medication.

Subjects may continue to receive treatment beyond confirmed progression in the absence of clinically significant deterioration and if investigators expect continual benefit from the treatment. For statistical analyses, these subjects will be considered to have investigator-assessed progressive disease at the time of the initial progression event.

#### Radiotherapy

Radiotherapy planning will be based on computed tomography (CT) images with minimal 5 mm slices. For robust and reproducible patient positioning during both planning and treatment, all positioning aids (e.g. masks, cushions, vacuum beds) are allowed.

For irradiation, tumor lesions in all non-cerebral/non-pulmonary locations can be included if radiotherapy is indicated and prescribed according to common good clinical practice (e.g. bone, soft tissue, lymph nodes). Internal organ metastases (such as in liver, pancreas, adrenal glands) or brain metastases should not be irradiated. Patients with thoracic lesions (e.g. thoracic spine, chest wall) or mediastinal lymph nodes will be included if the planning target volume (PTV) for irradiation does not directly intersect with the contoured lungs.

Gross tumor volumes (GTV) are contoured on the planning CT, considering additional co-registered imaging techniques such as magnetic resonance imaging (MRI) or positron emission tomography (PET), if available. Derived from the GTV, clinical (CTV) and planning target volumes (PTV) are created using common institutional margins to confidently cover radiation targets and simultaneously spare organs at risk (OAR). Contouring of relevant OAR is only necessary in cases of close vicinity or anticipated critical radiation exposure. In case of thoracic target volumes, both lungs will be contoured to monitor lung dose exposure, which is required to be as low as reasonably achievable. In cases of subtotal lung registration, the OAR “both partial lungs” will be generated.

Radiation delivery must be planned 3D-conformally and CT-based with either photons or electrons including step-and-shoot/helical/volumetric intensity-modulated, stereotactic or conventional techniques using linear accelerators. Radiotherapy is delivered on workdays in 5 single fractions of 4 Gy up to a total dose of 20 Gy. Therefore, radiotherapy is expected to last no longer than 2 weeks. Dose specification follows the requirements of the reports 50, 62, and 83 of the International Commission of Radiation Units and Measurements.

Adequate patient positioning and correct isocenter localisation are verified radiologically making use of either kV/MV-cone/fan-beam-CTs or conventional X-ray-documentation of either isocenter or radiation fields. If positioning corrections are necessary, they must be documented in the radiotherapy protocol.

#### Tissue and blood collection for exploratory endpoints

##### Tissue collection

For each patient a formalin-fixed, paraffin-embedded (FFPE) tumor tissue block (archival or recent) or a minimum of 15 unstained slides of tumor sample (2–3 μm sections; slices must be recent and collected on slides provided by the sponsor) must be available for biomarker (PD-L1) evaluation as stated in the inclusion criteria. Biopsy should be excisional, incisional or core-needle. Fine-needle aspiration is insufficient. Tumor PD-L1 assessment for retrospective sub-group analysis will be performed centrally according to institutional standards using the PD-L1 IHC 28–8 pharmDx assay.

It will be at the discretion of the investigator to determine whether a re-biopsy of a patient is required. The decision to re-biopsy shall be based on clinical judgment and necessity and should follow local guidelines and standards. If a recent biopsy has been collected and submitted, submission of archival tissue, if available, is still highly encouraged. In cases where retrospective haematoxylin and eosin staining by the central lab determines insufficient amounts of tumor tissue for the biomarker analyses, additional archived tissue may be requested by the sponsor, if available. If a re-biopsy after progression under study treatment is performed, submission of this tumor material is highly valued.

##### Blood collection

Participation of patients in the biomarker program is voluntary and must be documented in the informed consent form.

Routine haematological analysis of red and white blood cells and platelets are part of the scheduled patient assessments under therapy and are not a specific part of the exploratory biomarker program. However, their results are mandatory baseline information as absolute cell counts for further investigations.

Blood samples are planned to be taken in the context of study inclusion and before any therapeutic intervention, and serve as baseline controls. Furthermore, second blood samples are collected in group A on cycle 1, day 8 (visit 2) after completion of radiotherapy to assess early radiogenic immune response in combination with nivolumab. The second blood sample in group B is also collected on cycle 1, day 8 (visit 2) to assess early nivolumab-related immune response without radiotherapy. A third blood sample will be taken for both groups on cycle 3, day 1 (visit 4), when radiotherapy is completed in group A and when all patients (groups A and B) have received two doses of nivolumab. A fourth and final blood sample will be collected for both groups either on cycle 7, day 1 or upon end of treatment, whichever occurs first.

### Study endpoints

#### Primary endpoint

The primary endpoint will be the objective response rate (ORR) according to RECIST criteria 1.1.

#### Secondary endpoints

Secondary endpoints will be:

- PFS
- PFS and ORR using assessment according to irRECIST
- OS
- 1-year OS rate
- Descriptive sub-group analyses of efficacy in relation to PD-L1 expression levels (e.g. cut-off 1, 5, 10%)
- Treatment emergent adverse events according to common terminology criteria for adverse events (CTCAE) version 4.03
- Frequency of abnormal laboratory parameters
- Quality of Life [FACT-L, validated in [21]

#### Exploratory endpoints

Radiation-induced tumor-specific immune effects can explain events of tumor regression upon radiation treatment both within and beyond the irradiated fields and the immune system can be further stimulated by administration of a PD-1 blocking antibody such as nivolumab. The translational research accompanying this trial aims to elucidate the synergistic, immunostimulatory effects of radiotherapy and checkpoint inhibition that underlie these observations by covering the following aspects.

##### Exploratory analysis on radiation planning and dose administration

Exploratory analysis on radiation planning and dose administration will help to substantiate the phenomenon of radiation-induced abscopal effects. It will improve the option of radiation triggered systemic response through identification and possible prediction of both eligible targets for irradiation and probable lesion of abscopal response. To this end, both the location and composition of radiation targets and the anatomical profile of abscopally responding lesion must be carefully studied.

The following endpoints will address these questions:

Radiation oncology endpoints:

- Specific anatomical location of irradiated targets
- Specific characteristics and composition of irradiated targets (e.g. bone metastasis [met.] vs. soft connective tissue met. vs. soft parenchymatous tissue met. vs. lymph node met.)
- Absolute size of GTV, CTV and PTV
- Dose-volume histogram parameters such as D2%, D50%, and D98% within the PTV
- Radiation technique (e.g. 3D conventional vs. intensity-modulated radiotherapy) and beam energy

Radiology endpoints:

- Specific anatomical location of abscopally responding target lesions
- Specific characteristics and composition of abscopally responding target lesions (e.g. bone metastasis [met.] vs. soft connective tissue met. vs. soft parenchymatous tissue met. vs. lymph node met.)
- Characteristics of abscopal response (size reduction [RECIST] vs. tissue density vs. contrast enhancement/perfusion vs. other signs of response)

##### Exploratory analysis on tissue samples

Tumor PD-L1 assessments will be performed as part of the clinical study. The results will be used both retrospectively for patient sub-grouping and within the biomarker program for correlation analysis. The PD-L1 IHC 28–8 pharmDx assay will be used.

In addition, the immune infiltrate associated with the tumor will be analyzed to identity different subsets of immune cells. To this end, tissue slides will be subjected to a panel of IHC markers capable of identifying various types of immune cells. Depending on the availability of tumor material, tissue samples will also be analyzed with respect to microsatellite instability.

##### Exploratory analysis on blood samples

Blood samples that are collected at different time points will be used to characterize the immune response and investigate biological processes before, during and after the administration of the treatment. Briefly, phenotypic fluorescence activated cell sorter (FACS) analysis will be used to analyze whole blood samples with respect to the changes in the T-cell composition. Furthermore, specific T-cell responses to a panel of putative lung cancer-associated antigens will be measured by IFN-γ enzyme linked immunospot technique using frozen peripheral blood mononuclear cells. T-cell receptor sequencing will be performed for 2 visits per patient (baseline and end of study time point), in order to elucidate whether and, if so, which of the T-cell clones will be expanded during treatment. Abundances of immunostimulatory cytokines will be quantified by measuring serum cytokines. Finally, changes in lymphocyte gene signature arising from the synergistic treatment of patients with radiotherapy and nivolumab will be monitored by mRNA expression profiling using mRNA isolated from whole blood and using the human HT-12 v4 Expression BeadChip Kit.

### Statistical analysis

#### Sample size calculation

The primary endpoint will be the ORR according to RECIST criteria 1.1. Based on the results from the Checkmate 057 trial (ClinicalTrials.gov Identifier: NCT01673867; [5]), the ORR in group B is assumed to be 19%. Moreover, in the population with high PD-L1 expression (PD-L1 > 10%) an ORR of 37% was observed [5]. It is hypothesized that by combining nivolumab and radiotherapy an ORR of 35% can be achieved in both PD-L1-negative and -positive patients.

The study requires *n* = 50 subjects (in group A) to detect whether the responding proportion (ORR) is higher than 19% by applying a binomial test at a one-sided significance level of 0.05 with a probability of 1-beta = 0.8, assuming an actual response rate of 35%.

Due to the fact that a retrospective PD-L1 analysis will be crucial to reach the study objectives, the PD-L1 status should be available for at least 50 patients per treatment group. Practical experiences indicate that at a rate of 20–30% tumor tissue samples do not contain sufficient material for a PD-L1 IHC assessment. By taking this and also potential patient dropouts into account, the total number of patients enrolled will be *n* = 65 per group to ascertain the required sample number for the PD-L1 sub-group analysis and to achieve a sufficient statistical power for the primary analysis in case of patient dropouts.

#### Methods of statistical analysis

Statistical analysis is based on the International Conference on Harmonization Guidelines “Structure and Content of Clinical Study Reports” and “Statistical Principles for Clinical Trials”. The primary analysis set for all efficacy outcomes will be the intention-to-treat (ITT) population, while the per-protocol (PP) population will be used for sensitivity analyses.

The primary endpoint ORR will be evaluated by reporting absolute and relative frequencies for both treatment groups. For group A, a binomial test will be conducted at a one-sided significance level of α = 0.05 in order to assess if the ORR exceeds 19%. Furthermore, one-sided 95%-confidence intervals will be calculated for the ORR in both groups.

For the secondary time-to-event outcomes OS and PFS, median survival times and 1-year rates will be given with 95% confidence intervals and Kaplan-Meier curves will be calculated for both treatment groups.

Descriptive sub-group analyses with regard to PD-L1 status will be conducted assessing the primary and secondary outcomes separately for the patient strata PD-L1 high/low (e.g. cutoffs 1, 5, and 10%) by reporting the same statistical measures as described before for both treatment groups.

Safety analysis will be done for all patients who received at least one dose of study medication and according to the treatment actually received. It includes a tabulation of relative and absolute frequencies for adverse and serious adverse events. After 25 patients have been treated in group A, a descriptive safety report will be generated and evaluated by the Data Safety Monitoring Board.

### Trial status

The first patient was enrolled in March 2017. The FORCE trial is currently recruiting patients.

## Discussion

Lung cancer is the most common cause of cancer death worldwide with approximately 85% of patients suffering from NSCLC. Until recent years, treatment of NSCLC has been limited to chemotherapy with a sparse impact on median progression-free survival times ranging from 3 to 4 months. The therapeutic concept of restoring the patients’ antitumor immunity by inhibition of immune checkpoints such as PD-1/PD-L1 has been a groundbreaking advancement. Currently, PD-1/PD-L1-targeting checkpoint inhibition has even advanced up to 1st-line treatment, either as a monotherapy or in combination with chemotherapy. Despite this important progress a crucial remaining question is how T-cell-mediated antitumor immune responses upon checkpoint inhibition can be activated in non-responders, which account for approximately half of the patients [3, 4, 6, 22].

Palliative radiotherapy is a frequent therapeutic necessity in metastatic lung cancer. Preclinical studies, single patient reports, and retrospective analysis have shown that the combination of immunotherapy and radiotherapy can exert independent antineoplastic effects and simultaneously promote and perpetuate the radiation-associated abscopal effect [19, 23–25]. However, prospective studies investigating these observations are scarce. In a large phase III trial with patients with metastatic prostate cancer, no benefit of cytotoxic T-lymphocyte-associated antigen-4 (CTLA-4) blockade after radiotherapy was observed [26]. Consistent with these results, all reported preclinical and clinical cases of the combination have occurred with radiation therapy given either concomitantly or following CTLA-4 blockade [27]. Thus, it is a rational approach to prospectively investigate the potential benefit of PD-1/PD-L1 blockade concurrent with radiotherapy. This trial aims to recruit patients into group A with a clinical necessity for a radio-oncologic treatment. The appropriate palliation of metastatic disease is a governing treatment goal in these patients and must be performed with diligence. Therefore, no dose finding for the radiation dose will be performed since the risk of sub- or supratherapeutic dose regimens cannot be justified ethically or clinically. The FORCE trial will employ a radiotherapy regimen, which on its own is demonstrably efficacious and safe [28].

The fractionation regimen of 5 × 4 Gy (total dose 20 Gy) aligns with current standard-of-care practices. Compared to more hypofractionated regimens (i.e. less fractions at higher single doses), the FORCE regimen reduces the risk for re-irradiations to become necessary for symptom control (FORCE 10% vs other regimens 23%) [29]. Furthermore, higher single doses (i.e. 1 × 8 Gy) are associated with an increased incidence of pathologic bone fractures (3.2% vs. 2.8%) or spinal compression events (2.8% vs. 1.9%) [28].

The hypofractionated dose regimen chosen for radiotherapy in this trial is known to augment antigen presentation to cells of the anti-tumoral immune system and augment adaptive upregulation of PD-L1 by tumor cells [30, 31]. Compared to less hypofractionated regimens (e.g. 10 × 3 Gy) the FORCE regimen significantly reduces the treatment duration, hence allows to reach the treatment goal of local control of metastasis faster without compromising general patient safety.

Due to the scarcity of data from prospective clinical trials addressing radio-immunological treatments in NSCLC patients, it is difficult to predict the treatment-related toxicity. Especially for PD-1/PD-L1 targeting approaches, there are no clinical studies in lung cancer patients that provide reliable safety and toxicity data. Apart from mere toxicity, missing the optimal radiotherapy fractionation regime to be combined with pharmacological immunotherapy appears to be an issue of concern. A recent report about patients with metastatic solid tumors showed abscopal responses in as many as 22% of NSCLC patients when hypofractionated radiotherapy (single dose: 3.5 Gy) was combined with granulocyte-macrophage colony-stimulating factor based immunotherapy [24]. In this study, grade 3–4 toxicity occurred in about 20% of all patients. Additionally, one patient suffered from grade 4 pulmonary embolism. Van den Heuvel et al investigated the effect of irradiation of NSCLC metastasis in conjunction with an immune-stimulating fusion peptide (NHS-Interleukin-2) [32]. The radiation dose regimen was identical to the FORCE trial (5 × 4 Gy), and no toxicity was observed that could not be explained by the safety profile of Interleukin-2 (e.g. grade I-II thyroiditis in 25% of study subjects). Most clinical data about toxicity from combined modality regimes are derived from retrospective CTLA-4-targeting melanoma series and comprise autoimmune phenomena (e.g. hypophysitis) and – in case of cerebral radiotherapy – brain necroses [33–35]. In a retrospective study on patients with brain metastasis treated with radio-surgical ablative doses (1 x > 16 Gy) and concomitant PD-L1 immune-checkpoint blockade (nivolumab) no toxicities ≥ grade II were observed, although the brain is a radiosensitive organ [36]. Nevertheless, in the context of the FORCE trial irradiation of vulnerable organs such as brain and lung will not be permitted for safety reasons.

The significance of PD-L1 expression of the tumor is a contentious issue. Data from clinical trials indicate that efficacy may correlate with the PD-L1 expression in the primary tumor, whereas other studies report no correlation [9]. To date, marketing approval of nivolumab for squamous and non-squamous NSCLC does not mandate PD-L1 expression testing, whereas pembrolizumab monotherapy requires testing, and a companion diagnostic has been approved by the FDA. Therefore, further investigation of the role and impact of PD-L1 expression – incorporating the current knowledge – is warranted.

Closely connected to the PD-L1 expression are the underlying immune-biological mechanisms of PD-L1 targeting and the potential immune-stimulating effect of radiotherapy. To this end, a systematic exploration to better understand the immune-mediated tumor-host interaction – and thereby the potential impact of PD-1/PD-L1 antibody-treatment – in a true clinical setting is pivotal. The role of tumor-infiltrating lymphocytes and their functional contribution, the biological prerequisites for an immune-mediated tumor-host interaction, and the general notion of an “inflammatory preponderance” of the tissue surrounding the tumor are questions of immense interest to understand the effect of checkpoint inhibitor treatment [37, 38]. An extensive exploratory translational research program attached to this trial will focus on these potential mechanisms of action and the identification of potential biomarkers.

In summary, the rationale of the FORCE trial can be synthesized into three interconnected goals:

- to determine the safety and feasibility of the radio-immunological treatment approach;
- to increase PD-1 checkpoint inhibitor efficacy in metastatic non-squamous NSCLC by inducing an immune-sensitizing effect (abscopal-like effect) with radiotherapy;
- to explore the fundamental immunological principles that underlie checkpoint inhibitor efficacy and the immune-stimulating effect of radiotherapy in order to elucidate tumor-host biology and to find potential novel biomarkers.

## Data Availability

Not applicable – as no primary data are contained, generated or analysed.

## Abbreviations

AE: Adverse event
AIO: Arbeitsgemeinschaft Internistische Onkologie
CRF / eCRF: Case report form/electronic case report form
CT: Computed tomography
CTCAE: Common terminology criteria for adverse events
CTLA: T-lymphocyte-associated antigen
CTV: Clinical target volume
ECOG: Eastern Cooperative Oncology Group
EOT: End of treatment
FACS: Fluorescence activated cell sorter
FFPE: Formalin-fixed, paraffin-embedded
GTV: Gross tumor volumes
Gy: Gray
HBV: Hepatitis B virus
HCV: Hepatitis C virus
IHC: Immunohistochemistry
irRECIST: Immune-related response evaluation criteria in solid tumors
ITT: Intention-to-treat
MRI: Magnetic resonance imaging
NSCLC: Non-small cell lung cancer
OAR: Organs at risk
ORR: Objective response rate
OS: Overall survival
PD-1: Programmed death-1
PD-L1: Programmed death-ligand 1
PET: Positron emission tomography
PFS: Progression free survival
PP: Per-protocol population
PTV: Planning target volume
QoL: Quality of life
RECIST: Response evaluation criteria in solid tumors
SAE: Severe adverse event
